# Prostate cancer incidence and survival in relation to prostate cancer as second cancer in relatives

**DOI:** 10.1002/cam4.4591

**Published:** 2022-03-21

**Authors:** Guoqiao Zheng, Jan Sundquist, Kristina Sundquist, Jianguang Ji

**Affiliations:** ^1^ Center for Primary Health Care Research Lund University/Region Skåne Malmö Sweden; ^2^ Department of Family Medicine and Community Health, Department of Population Health Science and Policy Icahn School of Medicine at Mount Sinai New York USA; ^3^ Center for Community‐Based Healthcare Research and Education (CoHRE), Department of Functional Pathology, School of Medicine Shimane University Matsue Japan

**Keywords:** cancer screening, cumulative incidence, familial clustering, multiple primary cancer, prognosis

## Abstract

**Objectives:**

To investigate if the risk of prostate cancer (PC) differs based on the order of primary PC diagnosed in first‐degree relatives (FDRs) given possibly different risk factors for PC as first primary cancer (PCa‐1) and second primary cancer (PCa‐2).

**Subjects and Methods:**

In this Swedish nationwide cohort, PC diagnosis was followed for among 149,985 men with one FDR affected by PCa‐1, 10,972 with one FDR affected by PCa‐2 and 2,896,561 without any FDRs affected by cancer in a maximum of 57 years. PC patients were further followed for death due to PC since diagnosis. Relative risk (RR) of PC was estimated with Poisson regression and hazard ratio (HR) with Cox proportional hazard model.

**Results:**

Compared to men without any FDRs affected by cancer, the RRs of PC in men with one FDR affected by PCa‐1 and PCa‐2 were 2.12 (95% confidence interval [CI]: 2.07–2.17) and 1.69 (1.54–1.85), respectively. The risk in men with one FDR affected by PCa‐2 was significantly lower than those with one FDR affected by PCa‐1 after additionally adjusting for family relationship (father‐son and brothers) and age at diagnosis of PC in FDR (RR _PCa‐2 vs PCa‐1_, 0.85, 95% CI, 0.78–0.94). PC patients with a family history of PCa‐2 were more likely to be detected at late‐stage and less likely to be diagnosed by screening, compared to those with a family history of PCa‐1. Patients whose PC was diagnosed after the diagnosis of PCa‐1 in FDRs had a better survival than those without a family history of cancer (HR, 0.88, 95% CI, 0.80–0.97), but no such association was observed among patients with a family history of PCa‐2.

**Conclusion:**

Our study indicates a discrepancy between PC risks associated with a family history of PCa‐1 and PC‐2 and the reason behind it may be multifactorial.

## Graphical abstract

This is the first study considering risk of prostate cancer (PC) in association with PC diagnosed as first primary (PCa‐1) and second primary malignancy (PCa‐2), respectively, in first‐degree relatives. The PC risk was higher for men with family history of PCa‐1, compared to that for men with family history of PCa‐2. Family history of PCa‐2, unlike PCa‐1, was not associated with favorable survival. The finding provides evidence for PC prevention and screening for men with a family history of PC‐2.

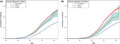

## 1 INTRODUCTION

Prostate cancer (PC) is the second most common cancer diagnosed in men and the leading cause of cancer‐related death worldwide.^1^ In the United States, 10‐year relative survival for localized PC was 100% during 2001–2016, while for the metastatic disease, 5‐year relative survival was only 30%.^2^ This emphasizes the importance of early detection among high‐risk groups. Older age, African ancestry, and family history are the only well‐established risk factors of PC, and thus are keys for risk assessment and screening.^3^, ^4^

PC diagnosis in a first‐degree relative (FDR) confers a 28% cumulative incidence in men by age 79 and a two‐fold relative risk (RR) compared to those without a family history.^5^ Current evidence regarding familial aggregation of PC is mainly based on the first primary PC (PCa‐1). With the increasing number of second primary cancers, the risk of PC associated with PC diagnosis as a second primary malignancy (PCa‐2) in FDRs remains uncharacterized. In addition to risk factors that predispose to PCa‐1, diagnosis of PCa‐2 may also be influenced by treatment and intensive medical surveillance in cancer patients which are not familial.^6^ Furthermore, the onset of PCa‐2 could happen at a much older age than that of PCa‐1, while early‐onset cancer is associated with higher familial risk in FDR than later‐onset ones.^5^, ^7^ In all, this points towards possible differences between family histories of PCa‐1 and PCa‐2, understanding which can help illuminate caveats in the risk‐adapted PC screening strategy.

PC patients with a family history (albeit PCa‐1) are shown to have better survival.^8^, ^9^ It is speculated to be due to the early clinical stage at diagnosis in patients with PC family history. Family history of PC is a major if not the most important factor for PC screening. Differentiating analyses of the tumor stage and survival outcomes among PC patients with a family history of PCa‐1 and PCa‐2 may tell us if FDRs of patients with PCa‐1 and PCa‐2 are screened similarly. Therefore, we aimed to assess PC risk and PC‐specific survival associated with a family history of PCa‐2 and compare it against that of PCa‐1.

## 2 SUBJECTS AND METHODS

### 2.1 Data resources

Data were derived from multiple Swedish national registers connected via a unique masked individual identification number. The Swedish Multi‐Generation Register is comprised of people born since 1932 or registered in the population registry since 1961. The biological parents were also present providing the family relationships. Established in 1958, the Swedish Cancer Register covers over 90% of all incident tumors in Sweden.^10^ The notification of cancer was based on the 7th version of the International Classification of Disease (ICD‐7) and updated using the subsequent latest versions. In Sweden, the diagnosis of multiple primary cancers follows the IARC/IACR multiple cancer coding rules,^11^ which is different from the rules set by the Surveillance, Epidemiology, and End Results (SEER) Program. For example, SEER takes the timing of the diagnoses into consideration, whereas according to IARC rules, recognition of the existence of two or more primary cancers does not depend on time.^12^ However, in this study, we defined PCs diagnosed at least 1 month after first primary non‐PC cancer as PCa‐2. Cause of death was retrieved from the Cause of Death Register. Information on socioeconomic status and place of residence was obtained by further linkage to the Total Population Register.

### 2.2 PC risk associated with a family history of PCa‐1 and PCa‐2

The design of the familial risk analysis is shown with an example in Figure S1. Family history of PCa‐2 was defined as PC diagnosis after other first primary cancer in FDR (father or brother); as shown in Figure S1A, the father was diagnosed with PC at age2 after cancer A. Family history of PCa‐1 was defined as single PC diagnosis in FDR (Figure S1B). Men without cancer diagnosis in FDR were used as a reference group (Figure S1C). Approximately 4.3 million men were identified at risk of PC in the offspring generation. We excluded men: (1) with FDRs affected by multiple primary PCs as it is difficult to differentiate a new tumor from recurrence in the same organ, (2) with FDRs affected by higher‐order (third, fourth, etc.) multiple primary cancers, (3) with more than one FDR affected by cancers and (4) with FDRs affected by other cancer. A total of 2,896,561 men were without FDRs affected by cancer (reference), 149,985 with a family history of PCa‐1 and 10,972 with a family history of PCa‐2. They were followed from 1958, year of birth or immigration, whichever came latest, to 2015, year of PC diagnosis, death or migration, whichever came earliest. In order to assess the possible bias due to the exclusion of the men in any of the above conditions, we conducted a sensitivity analysis including all the men in the offspring generation. They were classified into five groups based on PC diagnosis in FDRs: No PCa‐1 or PCa‐2 (*N* = 3,973,703), only PCa‐1 (*N* = 273,149), only PCa‐2 (*N* = 20,130), both PCa‐1 and PCa‐2 (*N* = 1712), and multiple primary PCs (*N* = 262).

The RRs of PC were estimated with Poisson regression using men without a family history as the reference. In the sensitivity analysis, men without a family history of PCa‐1 or PCa‐2 were used as a reference population. Age groups (5 years), periods (5 years), socioeconomic status (blue‐collar worker, white‐collar worker, farmer, private business, professional, or other/unspecified) and place of residence (big cities, northern Sweden, southern Sweden, and unspecific) were additionally adjusted for. We further stratified the risk based on the age (≤65 and >65) at diagnosis of PC in FDRs, type of family relationships (father‐son and brothers), time interval (i.e., age2 – age1 in Figure S1A) between first primary cancer and PCa‐2 in relatives, and sites of first primary cancer (cancer A in Figure S1A) before PCa‐2. The purpose to stratify the site of the first primary cancer was to give a more precise PC risk estimation, as different first primary cancers have specific genetic and environmental risk factors and cancer treatment that may be associated with the development and/or diagnosis of PC. Comparison between familial risks in men with a family history of PCa‐1 and PCa‐2 was explored additionally by adjusting age at diagnosis of PC in FDRs (as a continuous variable) and family relationship. We calculated the cumulative incidence and the 95% confidence interval (CI) from birth to a specific age considering death and diagnosis of other cancer as competing events.

### 2.3 PC‐specific survival associated with a family history of PCa‐1 and PCa‐2

For the PC‐specific survival, all the PC patients diagnosed in the previous risk estimation analysis (gray squares in offspring generation in Figure S1) were considered. We only included patients whose PCs were diagnosed after PC diagnosis in FDR, as family members are more likely to seek PC screening after the appearance of first PC in the family. They were followed from the year at diagnosis of PC until December 2015. Death due to other causes were censored.

With Cox regression, we estimated the hazard ratio (HR) of death due to PC by using PC patients without cancer family history as the reference group. We adjusted for age and year of diagnosis of PC, socioeconomic status, place of residence, and clinical stage. Clinical stage was classified into 0, I, II, III, IV based on TNM status through the AJCC convention, 8th version.^13^ As the application of TNM staging system in the register started since 2003, so PC cases that were diagnosed before had missing information on staging. Among patients who had TNM data, patients with undefined T, N, and M (coded as Tx, Nx and Mx in the cancer registry) were grouped separately if they were unable to meet any stage classification. In Sweden, tumor size (T) for screening‐detected PC was recorded as T1c. We compared the proportion of screening‐detected PC with different PC family histories with Chi‐square test.

A two‐tailed *p*‐value of <5% was considered significant. All the statistical analyses were done in SAS 9.4 version.

## 3 RESULTS

### 3.1 Familial risk of PC stratified by age of PC diagnosis in FDRs and family relationship

**FIGURE 1:**
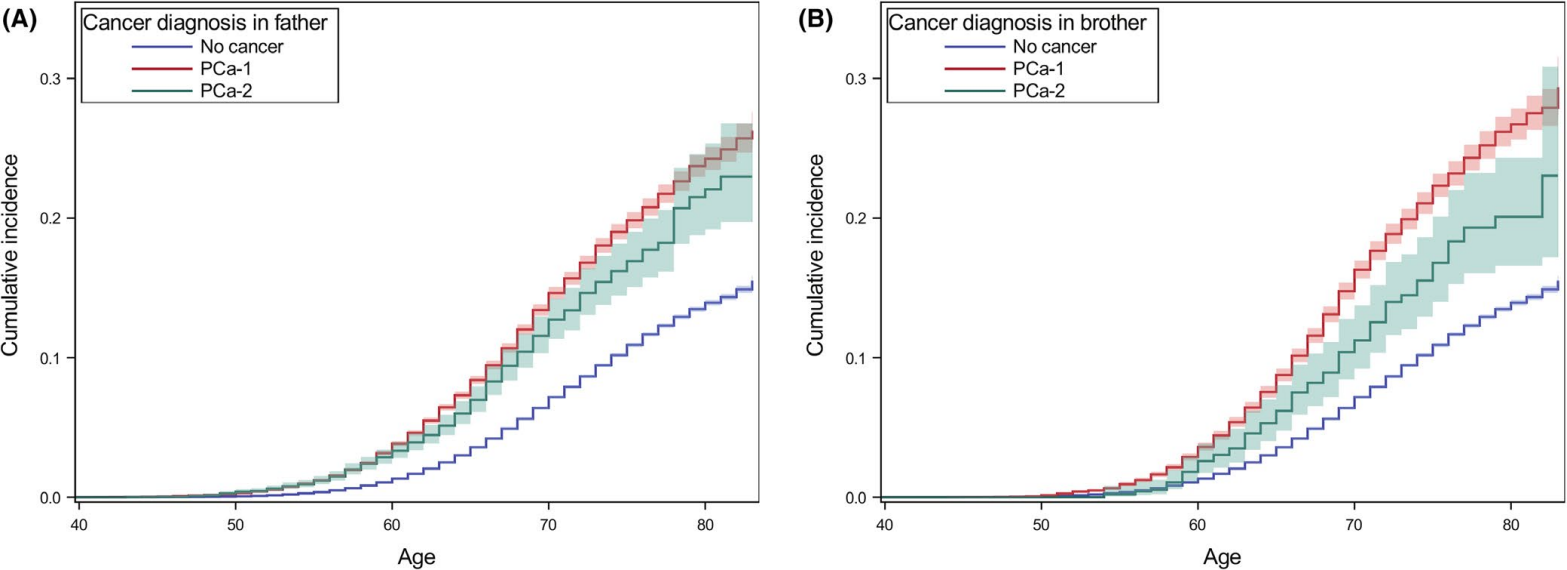
Cumulative incidence of prostate cancer in men with a family history of PCa‐1 or PCa‐2 in father (A) and brother (B). PCa‐1, prostate cancer as a first primary malignancy, PCa‐2, prostate cancer as a second primary malignancy. The shading band is the 95% confidence interval of the cumulative incidence. PCa‐1, prostate cancer as a first primary malignancy; PCa‐2, prostate cancer as a second primary malignancy

The median (interquartile range, IQR) age at diagnosis of PCa‐2 in FDRs was 75 (69–81) years, higher than PCa‐1 (72, 66–79, *p* < 0.0001) diagnosed in FDRs. The median ages at diagnosis of PC in the offspring generation with either family history of PCa‐1 or PCa‐2 were the same, 64 years (59–69). RR (95% CI) of PC with a family history of PCa‐1 was 2.12 (2.07–2.17), higher than that for PCa‐2 (1.69, 1.54–1.85) (Table 1). This risk was higher if the family member was diagnosed with PC before age 66. While considering the family relationship, the risk increased when a sibling had PCa‐1 (2.26, 2.17–2.35). Whereas for PCa‐2, the paternal (1.72, 1.56–1.91) and fraternal familial risks (1.60, 1.34–1.91) were similar. The risk with a family history of PCa‐2 was significantly lower than that with PCa‐1 after additional adjustments for age at diagnosis of PC in FDRs and family relationship (overall RR, 0.85, 95% CI, 0.78–0.94). For paternal family history of a PCa‐1 and a PCa‐2, the cumulative incidence of PC (95% CI) by age 80 was 27.4% (26.5%–28.4%) and 25.1% (21.6%–29.2%), respectively (Figure 1). For a fraternal family history, the corresponding cumulative incidences were 30.1% (28.8%–31.5%) and 22.4% (18.4%–27.4%). In the sensitivity analysis (Table S1), the RR for family history of PCa‐1 (2.29, 2.26–2.33) was higher than that for PCa‐2 (1.77, 1.67–1.87), which was consistent in the main analysis (Table 1). A much higher familial risk was observed among men with FDRs affected by both PCa‐1 and PCa‐2 as well as men with FDR affected by multiple primary PCs.

**TABLE 1:** Prostate cancer risks stratified by family history of PCa‐1 and PCa‐2 among offspring generation

Category	Family history of PCa‐1^a^			Family history of PCa‐2^a^			PCa‐2 vs. PCa‐1^b^	
	No. of PC	RR	95% CI	No. of PC	RR	95% CI	RR	95% CI
Overall	8105	2.12	2.07–2.17	497	1.69	1.54–1.85	0.85	0.78–0.94
Diagnostic age of PC in FDR								
≤65 years old	2061	2.82	2.69–2.94	61	1.99	1.55–2.59	0.70	0.55–0.91
>65 years old	6044	1.95	1.90–2.01	436	1.66	1.51–1.82	0.87	0.80–0.96
Family relationship								
Father‐son	5410	2.05	1.99–2.11	377	1.72	1.56–1.91	0.88	0.79–0.98
Brothers	2695	2.26	2.17–2.35	120	1.60	1.34–1.91	0.80	0.67–0.96

Abbreviations: CI, confidence interval; FDR, first‐degree relative; PC, prostate cancer; PCa‐1, prostate cancer as a first primary malignancy; PCa‐2, prostate cancer as a second primary malignancy; RR, relative risk.

^a^ RR was estimated from Poisson regression using individuals without cancer family history as the reference. The covariates adjusted in the model included age groups (5 years), periods (5 years), socioeconomic status (blue‐collar worker, white‐collar worker, farmer, private business, professional, or other/unspecified), and place of residence (big cities, northern Sweden, southern Sweden and unspecific).

^b^ Comparison between risks of PC associated with a family history of PCa‐2 and PCa‐1. Men with a family history of PCa‐1 were used as the reference group. RR was estimated with additional adjustment on age at diagnosis of PC in FDR (as a continuous variable) and family relationship (father‐son and brothers).

### 3.2 Familial risk of PC stratified by site of the first primary cancer in FDR and, years between first primary cancer and PCa‐2 in FDR

The risk of PC for family history of PCa‐2 with stratification on first primary cancer is displayed in Table 2. Significant RRs were observed in cancers of upper aerodigestive tract (UAT), small intestine, colorectum, lung, breast, testis, kidney, bladder, skin, nervous system, thyroid, connective tissue, non‐Hodgkin lymphoma (NHL), myeloma, and leukemia. The highest risk was in the small intestine (3.37, 1.40–8.11), followed by breast (3.30, 1.06–10.23), UAT (2.52, 1.83–3.46), and testicle (2.59, 1.23–5.43) cancers. The median time from a first primary cancer to PCa‐2 diagnosed in FDR was 61 (16–144) months. For a better interpretation, we stratified the familial risk for men with FDR diagnosed with PCa‐2 within the first year, 2–5 years, 6–10 years, and over 10 years after the first primary cancer (Table S2, cancer sites with ≥15 PC cases were included). Despite no specific trend, the familial risks were relatively low, if PCa‐2 was diagnosed shortly after, especially within a year of the first primary colorectal, kidney or bladder cancer or NHL.

**TABLE 2:** Prostate cancer risk for men with a family history of PCa‐2 with stratification on sites of first primary cancer diagnosed in first‐degree relatives

First primary cancer site in FDR	No. of PC	RR^a^	95% CI
UAT	38	**2.52**	1.83–3.46
Esophagus	2	2.07	0.52–8.27
Stomach	10	1.08	0.58–2.01
Small intestine	5	**3.37**	1.40–8.11
Colorectum	101	**1.77**	1.46–2.15
Liver	4	1.52	0.57–4.04
Pancreas	2	1.19	0.30–4.77
Nose	1	0.58	0.08–4.13
Lung	27	**1.92**	1.32–2.80
Breast	3	**3.30**	1.06–10.23
Testis	7	**2.59**	1.23–5.43
Male genital	2	0.84	0.21–3.37
Kidney	30	**2.10**	1.47–3.01
Bladder	84	**1.31**	1.06–1.62
Melanoma	26	1.27	0.86–1.86
Skin	56	**1.81**	1.39–2.35
Eye	4	2.21	0.83–5.90
Nervous system	13	**1.80**	1.04–3.10
Thyroid	8	**2.26**	1.13–4.52
Endocrine gland	10	1.66	0.89–3.09
Connective tissue	12	**2.47**	1.40–4.35
NHL	21	**1.91**	1.25–2.93
Hodgkin lymphoma	2	1.01	0.25–4.04
Myeloma	7	**2.34**	1.12–4.91
Leukemia	19	**1.84**	1.17–2.89
CUP	3	1.32	0.43–4.09

*Note*: Significant RRs are in bold.

Abbreviations: CI, confidence interval; CUP, cancer of unknown primary; FDR, first‐degree relative; NHL, non‐Hodgkin lymphoma; PC, prostate cancer; PCa‐2, prostate cancer as a second primary malignancy; RR, relative risk; UAT, upper aerodigestive tract.

^a^ RR was estimated from Poisson regression using individuals without cancer family history as the reference. The covariates adjusted in the model included age groups (5 years), periods (5 years), socioeconomic status (blue‐collar worker, white‐collar worker, farmer, private business, professional, or other/unspecified) and place of residence (big cities, northern Sweden, southern Sweden and unspecific).

### 3.3 PC‐specific survival stratified by family history of PCa‐1 and PCa‐2

Among all the familial PC patients who were diagnosed after PC diagnosis in FDR, more patients with a family history of PCa‐2 were diagnosed at an advanced stage (stage III or IV, 18.6%) compared to those with a family history of PCa‐1 (14.3%, Table S3). Fewer patients with a family history of PCa‐2 were screening‐detected (47.6%) than those with a family history of PCa‐1 (50.3%). We further grouped PC cases based on the age at diagnosis and we found the difference in stage at diagnosis and screening was mainly for those diagnosed after age 65 (Table S3). Compared to patients without cancer family history (Table 3), as expected, those with a family history of PCa‐1 had a favorable PC‐specific survival (0.88, 0.80–0.97). This survival difference was observed only for younger cases (≤ 65 years). While for patients with a family history of PCa‐2, the survival was worse although not significant, notably for those older patients (1.50, 0.94–2.39).

**TABLE 3:** PC‐specific survival stratified by family history of PCa‐1 and PCa‐2 among prostate cancer patients whose PC was diagnosed after PC diagnosis in FDRs

Cancer in FDR	All PCs			PCs diagnosed ≤65 years			PCs diagnosed >65 years		
	No. of PCs	No. of death	HR (95% CI)	No. of PCs	No. of death	HR (95% CI)	No. of PCs	No. of death	HR (95% CI)
No cancer	26,330	2602	1.00	12,478	1400	1.00	13,852	1202	1.00
PCa‐1	6477	478	0.88 (0.80–0.97)	3636	269	0.86 (0.75–0.98)	2841	209	0.93 (0.80–1.07)
PCa‐2	397	33	1.09 (0.77–1.53)	224	15	0.82 (0.49–1.36)	173	18	1.50 (0.94–2.39)

*Note*: HR was estimated from Cox proportional regression using individuals without cancer family history as the reference. The covariates adjusted in the model included age, year and stage at diagnosis of PC, socioeconomic status (blue‐collar worker, white‐collar worker, farmer, private business, professional, or other/unspecified) and place of residence (big cities, northern Sweden, southern Sweden and unspecific).

Abbreviations: CI, confidence interval; FDR, first‐degree relative; HR, hazard ratio; PC, prostate cancer; PCa‐1, prostate cancer as a first primary malignancy; PCa‐2, prostate cancer as a second primary malignancy.

## 4 DISCUSSION

This nationwide cohort study showed that compared to family history of PCa‐1, the overall familial risk conferred by PCa‐2 is smaller, but a family history of PCa‐2 may confer an unfavorable PC‐specific survival, compared to those with a family history of PCa‐1. This indicates that the order of PC (at least first and second) diagnosed in relatives should be differentiated when considering the family history for PC screening.

Early‐onset PC has been associated with a greater hereditary background.^14^ The lower familial risk linked with a family history of PCa‐2 could be attributed to the older age at diagnosis of PCa‐2 in FDR than that of PCa‐1. With adjusting for age at PC diagnosis in FDR and family relationship, the difference remained significant suggesting other factors may contribute to the lower familial risk. The magnitude of the increased PC risk for men with a family history of PC could reportedly be inflated by familial aggregation of “PSA detected,” clinically insignificant, low‐risk PC.^15^ This corroborates the detection of more screening‐detected and less late‐stage PC in patients with a family history of PCa‐1. In the site‐specific analysis, the first primary bladder cancer in FDR accounted for the second most familial PC cases (*N* = 84) and was associated with a lower familial risk of PC. This could be due to incidental PCs detected among bladder cancer patients undergoing radical cystoprostatectomy, some of which could be clinically insignificant,^16^ and family history of nonfatal PC has been reported with a significantly lower familial risk compared to that with a family history of fatal PC.^17^ Similarly, the intense surveillance on nearby organs could contribute to the low familial risk when FDR developed PCa‐2 within a year of first primary kidney and colorectal cancer diagnosis.^18^, ^19^ The high familial risk for first primary UAT, small intestine, and kidney cancers could be associated with highly familial Lynch syndrome, or for breast cancer, HBOC syndrome.^20^

Despite the controversy regarding PC screening in the general male population, there is no doubt that individuals with a family history of PC benefit from it. We found that PC patients with a family history of PCa‐1 had a relatively smaller proportion of an advanced stage disease and a larger proportion of them were detected by screening compared to those with a familial PCa‐2. The early diagnosis of PC in patients with familial PCa‐1 could associate to the better survival as has been reported elsewhere.^21^, ^22^ Despite adjustment for clinical staging, the survival for PC patients with a family history of PCa‐1 was still better than those without cancer family history, particularly for patients whose PCs were identified after PC diagnosis in FDR. However, survival worsened those with a family history of PCa‐2, which may be attributed to a greater mutational burden from two cancers resulting in a more aggressive disease due to polygenic inheritance.^4^

By combining Swedish national registers, we assessed the familial risks with adequate statistical power and accurate family relationship in a nationwide population‐based setting. The results boast a high degree of validity of second primary cancers as an ad hoc study showed 98% diagnostic accuracy of second neoplasms in the registry and none were found to be a metastasis.^23^ We acknowledge that by retaining men with single FDR with PC, many families with possible cancer syndrome were filtered out. This was to control the effect from other cancer(s) present in the pedigree.^24^ In addition, individuals from families with multiple cancer patients are more likely to get increased medical attention than single FDR with PCa‐2. In the sensitivity analysis including all the men in the offspring generation, the familial risk for PCa‐1 and PCa‐2 showed a similar pattern as in the main analysis, which shows no evidence of bias in our results. As for the limitations, data on some risk factors such as diet, physical activity, smoking, and alcohol consumption were unavailable although adjustment for socio‐economic status was included as a proxy to reduce possible confounding.^25^, ^26^ Information on treatment for the first primary cancer in FDRs as well as any genetic data was lacking, which could have provided better explanations for associations with some specific first primary cancers. The findings from this study are confined to Sweden and further generalization requires careful consideration.

## 5 CONCLUSIONS

We found a lower familial risk associated with a family history of PCa‐2 compared to that with a family history of PCa‐1. We speculate three possible reasons: (1) late onset of PCa‐2 than that of PCa‐1 in FDR, (2) greater PC screening on men with a family history of PCa‐1 than with a family history of PCa‐2, and (3) intense medical surveillance on nearby organs among cancer patients, that leads to family history of clinically insignificant PCa‐2. The stage‐adjusted PC‐survival analysis further indicates that the order of the primary PC diagnosis in FDR should be considered when evaluating family history of PC which may benefit familial risk estimation and PC patient management.

## CONFLICT OF INTEREST

The authors have declared no conflicts of interest.

## AUTHOR CONTRIBUTIONS

Design: Jianguang Ji, Guoqiao Zheng. Acquisition of data: Jan Sundquist, Kristina Sundquist. Statistical analysis and interpretation: Guoqiao Zheng, Jianguang Ji, Jan Sundquist, Kristina Sundquist. Manuscript writing: Guoqiao Zheng and all other authors. Approval of the final text: All authors.

## ETHICAL APPROVAL AND PATIENT CONSENT

We secured ethical approval (6 February 2013) for this study from the Regional Ethical Review Board of Lund University (Dnr 2012/795). Patient consent was not required as the study used only de‐identified registry based secondary data.

## Supporting information


Figure S1
Table S1
Table S2
Table S3

## Data Availability

The data that support the findings of this study are available from Lund University but restrictions apply to the availability of these data, which were used under license for the current study and so are not publicly available.
