# Supplementary material for: Prostate cancer incidence and survival in relation to prostate cancer as second cancer in relatives
Source: Cancer Med. 2022 Mar 21;11(10):2117–24. doi: 10.1002/cam4.4591 (PMC9119351; doi:10.1002/cam4.4591)
Supplement: Supplementary file 1 — Figure S1 Table S1 Table S2 Table S3 [file CAM4-11-2117-s001.docx]

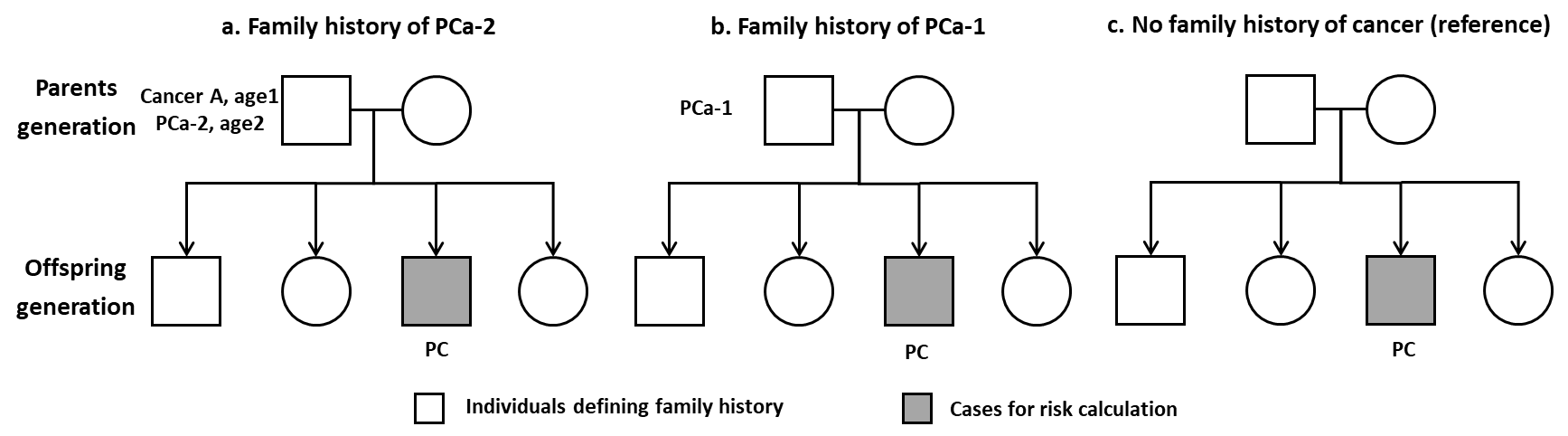


**Supplementary Figure 1.** Example of a pedigree for the analysis. Parents and siblings were used to define family history. Cases in the offspring generation were used to estimate risk. In Supplementary Figure 1a, father was first diagnosed with cancer A (first primary cancer) at age1 and then PCa-2 at age2. In Supplementary Figure 1b, father was diagnosed with PCa-1. In Supplementary Figure 1c, no first-degree relatives were diagnosed with any cancer. PC, prostate cancer, cancer A, any cancer other than prostate cancer, PCa-1, prostate cancer as a first primary malignancy, PCa-2, prostate cancer as a second primary malignancy.

**Supplementary Table 1.** Sensitivity analysis for association of PC risk and family history of PC among all the men in the offspring generation

| PC diagnosis in FDR | No. of PC | RR ^a^ | 95%CI |
| --- | --- | --- | --- |
| No PCa-1 and PCa-2 | 60972 | 1.00 | - |
| Only PCa-1 | 21067 | 2.29 | 2.26-2.33 |
| Only PCa-2 | 1238 | 1.77 | 1.67-1.87 |
| Both PCa-1 and PCa-2 | 460 | 4.44 | 4.05-4.87 |
| Multiple primary PC | 26 | 3.14 | 2.13-4.61 |

a, RR was estimated from Poisson regression using individuals without family history of PCa-1 and PCa-2 as the reference. The covariates adjusted in the model included age groups (5 years), periods (5 years), socioeconomic status (blue-collar worker, white-collar worker, farmer, private business, professional, or other/unspecified) and place of residence (big cities, northern Sweden, southern Sweden and unspecific).

PC, prostate cancer, PCa-1, prostate cancer as a first primary malignancy, PCa-2, prostate cancer as a second primary malignancy, FDR, first-degree relative

**Supplementary Table 2.** Prostate cancer risk stratified by site of first primary cancer and period between first primary cancer and PCa-2 in first-degree relatives among men with a family history of PCa-2

| First primary cancer site in FDR | **Period between first primary cancer and PCa-2 in FDR** | | | | | | | | | | | | | | | |
| --- | --- | --- | --- | --- | --- | --- | --- | --- | --- | --- | --- | --- | --- | --- | --- | --- |
|  | **≤ 1 year** | | | | **2-5 years** | | | | **6-10 years** | | | | **> 10 years** | | | |
|  | N | RR | 95%CI | | N | RR | 95%CI | | N | RR | 95%CI | | N | RR | 95%CI | |
| UAT | 13 | **6.40** | 3.72 | 11.0 | 8 | **2.18** | 1.09 | 4.35 | 5 | 1.44 | 0.60 | 3.47 | 12 | **2.02** | 1.15 | 3.57 |
| Colorectum | 15 | 1.29 | 0.77 | 2.13 | 39 | **2.30** | 1.68 | 3.15 | 22 | **1.63** | 1.08 | 2.48 | 25 | **1.68** | 1.14 | 2.49 |
| Lung | 13 | **2.01** | 1.17 | 3.47 | 6 | 1.58 | 0.71 | 3.52 | 4 | 2.39 | 0.90 | 6.37 | 4 | 1.84 | 0.69 | 4.89 |
| Kidney | 3 | 0.98 | 0.32 | 3.05 | 2 | 0.78 | 0.20 | 3.13 | 11 | **2.74** | 1.52 | 4.95 | 14 | **3.02** | 1.79 | 5.10 |
| Bladder | 26 | 1.07 | 0.73 | 1.57 | 23 | **1.59** | 1.06 | 2.40 | 15 | 1.35 | 0.82 | 2.24 | 20 | 1.41 | 0.91 | 2.19 |
| Melanoma | 2 | 1.13 | 0.28 | 4.54 | 5 | 0.99 | 0.41 | 2.38 | 4 | 1.10 | 0.41 | 2.92 | 15 | 1.52 | 0.91 | 2.51 |
| Skin | 10 | **2.30** | 1.24 | 4.27 | 26 | **2.28** | 1.55 | 3.34 | 9 | 1.33 | 0.69 | 2.55 | 11 | 1.33 | 0.74 | 2.40 |
| NHL | 1 | 0.88 | 0.12 | 6.23 | 5 | 1.48 | 0.61 | 3.55 | 7 | **2.91** | 1.39 | 6.11 | 8 | **2.03** | 1.01 | 4.05 |
| Leukemia | 3 | 2.13 | 0.69 | 6.62 | 8 | **2.33** | 1.16 | 4.65 | 6 | 1.73 | 0.78 | 3.86 | 2 | 1.03 | 0.26 | 4.12 |
| All | 126 | **1.65** | 1.39 | 1.97 | 125 | **1.82** | 1.53 | 2.17 | 120 | **1.55** | 1.29 | 1.85 | 126 | **1.79** | 1.50 | 2.13 |

UAT, upper aerodigestive tract, NHL, non-Hodgkin lymphoma, PCa-2, prostate cancer as a second primary malignancy, FDR, first-degree relative

**Supplementary Table 3.** Distribution of clinical stage and proportion of screening-detected prostate cancer in all prostate cancer patients whose prostate cancer was diagnosed after prostate cancer diagnosis in FDR

| Groups | All PCs | | | PCs diagnosed ≤65 years | | | PCs diagnosed > 65 years | | |
| --- | --- | --- | --- | --- | --- | --- | --- | --- | --- |
|  | Cancer diagnosis in FDR | | | Cancer diagnosis in FDR | | | Cancer diagnosis in FDR | | |
|  | No cancer | PCa-1 | PCa-2 | No cancer | PCa-1 | PCa-2 | No cancer | PCa-1 | PCa-2 |
|  | N (%) | N (%) | N (%) |  | N (%) | N (%) |  | N (%) | N (%) |
| **Stage** |  |  |  |  |  |  |  |  |  |
| 0 | 107(0.4) | 26(0.4) | 1(0.2) | 58(0.5) | 19(0.5) | 0 | 49 (0.4) | 7(0.2) | 1(0.6) |
| I | 11881(45.1) | 3199(49.3) | 191(48.1) | 5715(45.8) | 1877(51.6) | 118(52.7) | 6166 (44.5) | 1322(46.5) | 73(42.2) |
| II | 6268(23.8) | 1547(23.9) | 83(20.9) | 2551(20.4) | 764(21.0) | 36(16.1) | 3717(26.8) | 783(27.6) | 47(27.2) |
| III | 2561(9.7) | 570(8.8) | 48(12.1) | 802(6.4) | 213(5.9) | 14(6.2) | 1759(12.7) | 357(12.6) | 34(19.6) |
| IV | 1778(6.8) | 358(5.5) | 26(6.5) | 667(5.4) | 152(4.2) | 19(8.5) | 1111(8.0) | 206(7.2) | 7(4.0) |
| Undefined | 355(1.4) | 88(1.4) | 10(2.5) | 143(1.2) | 49(1.4) | 5(2.2) | 212(1.5) | 39(1.4) | 5(2.9) |
| Missing | 3380(12.8) | 689(10.6) | 38(9.6) | 2542(20.4) | 562(15.5) | 32(14.3) | 838(6.0) | 127(4.5) | 6(3.5) |
| Total | 26330(100) | 6477(100) | 397(100) | 12478(100) | 3636(100) | 224(100) | 13852(100) | 2841(100) | 173(100) |
| Chi-square P | <0.0001 | | | <0.0001 | | | 0.0029 | | |
|  |  |  |  |  |  |  |  |  |  |
| **Screening-detected PC** | |  |  |  |  |  |  |  |  |
| No | 12510 (54.5) | 2876 (49.7) | 188 (52.4) | 4859(48.9) | 1357(44.1) | 87(45.3) | 7651 (58.8) | 1519(56.0) | 101(60.5) |
| Yes | 10440 (45.5) | 2912 (50.3) | 171 (47.6) | 5077(50.1) | 1717(55.9) | 105(54.7) | 5363(41.2) | 1195(44.0) | 66(39.5) |
| Total | 22950 (100) | 5788 (100) | 359 (100) | 9936(100) | 3074(100) | 192(100) | 13014(100) | 2714(100) | 167(100) |
| Chi-square P | <0.0001 | | | <0.0001 | | | 0.0215 | | |

PC, prostate cancer, FDR, first-degree relative, PCa-1, prostate cancer as a first primary malignancy, PCa-2, prostate cancer as a second primary malignancy
